# A comprehensive mechanism of biological and health effects of anthropogenic extremely low frequency and wireless communication electromagnetic fields

**DOI:** 10.3389/fpubh.2025.1585441

**Published:** 2025-06-04

**Authors:** Dimitris J. Panagopoulos, Igor Yakymenko, Geoffry N. De Iuliis, George P. Chrousos

**Affiliations:** ^1^Choremeion Research Laboratory, 1st Department of Paediatrics, Medical School, National and Kapodistrian University of Athens, Athens, Greece; ^2^Electromagnetic Field-Biophysics Research Laboratory, Athens, Greece; ^3^Department of Ecology and Ecomanagement, National University of Food Technologies, Kyiv, Ukraine; ^4^Reproductive Science Group, School of Environmental and Life Sciences, College of Engineering, Science and Environment, University of Newcastle, Callaghan, NSW, Australia; ^5^University Research Institute of Maternal and Child Health and Precision Medicine and UNESCO Chair on Adolescent Health Care, National and Kapodistrian University of Athens, Medical School, Aghia Sophia Children’s Hospital, Athens, Greece

**Keywords:** electromagnetic fields, voltage-gated ion channels, oxidative stress, ion forced oscillation, IFO-VGIC mechanism, ROS, DNA damage

## Abstract

Exposure to anthropogenic electromagnetic fields (EMFs), especially those of wireless communications (WC) has increased tremendously. This is an unprecedented phenomenon throughout biological evolution because, all anthropogenic EMFs, being fully polarized, coherent, and, especially WC EMFs, highly variable, differ substantially from the natural EMFs. WC EMFs consist of Microwave (MW) carrier waves, modulated, by Extremely Low Frequency (ELF) signals, and included in on/off pulses repeated at various ELF rates. Moreover, they exhibit intense random variability, mainly in the Ultra Low Frequency (ULF) band. Thus, WC EMFs are a combination of MW and ELF/ULF EMFs. The combination of polarization/coherence and intense low-frequency (ELF/ULF) variability seems to be the key to EMF-bioactivity. Epidemiological and laboratory studies highlight a connection between ELF or WC EMF exposure and cancer, infertility, electro-hypersensitivity, and various other pathologies. Studies also find DNA damage and Oxidative Stress (OS) which explain these pathologies. While man-made EMFs cannot directly ionize molecules, they are capable of doing this indirectly in biological tissue, by triggering the biosynthesis of Reactive Oxygen Species (ROS) which can damage biomolecules, including DNA. The (over)production of ROS and the consequent OS are triggered by irregular gating of Voltage-Gated Ion Channels (VGICs) in the cell membranes as described by the Ion Forced Oscillation (IFO)-VGIC mechanism: Mobile ions within VGICs forced to oscillate by the applied ELF/ULF EMFs exert forces on the voltage sensors of the VGICs, similar to or greater than the forces that physiologically gate those channels, resulting in their irregular gating (dysfunction). Dysfunction of ion channels disrupts intracellular ionic concentrations. This triggers ROS overproduction and OS by the ROS-generating systems/enzymes in the cells, such as the electron transport chain (ETC) in the mitochondria, or the NADPH/NADH oxidases (NOXs), the Nitric Oxide synthases (NOS), etc. The IFO-VGIC mechanism and the consequent OS constitute a comprehensive mechanism that explains all known adverse biological and health effects reported to be induced by anthropogenic EMFs.

## 1 Introduction

### 1.1 Unique physical properties of anthropogenic and especially WC EMFs: Polarization/coherence, combination of frequency bands, modulation, pulsation, variability

**Figure 1:**
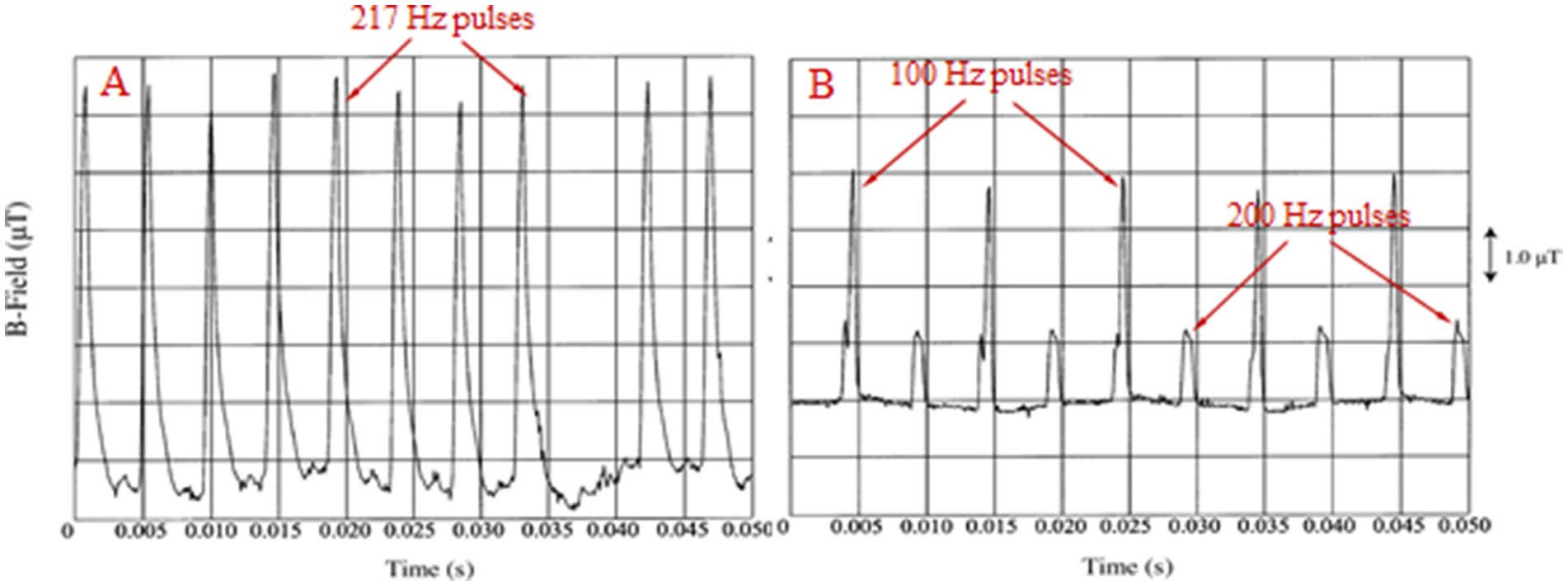
**(A)** 217 Hz (“frame repetition”) pulses from a GSM mobile phone. **(B)** 100 and 200 Hz pulses from a cordless domestic (DECT) phone [adapted from Pedersen (4)].

All man-made electromagnetic fields (EMFs) and corresponding electromagnetic radiation (EMR) are fully polarized and coherent as they are produced by electric/electronic circuits/antennas with specific geometrical shapes. Moreover, most anthropogenic EMFs and especially those generated by Wireless Communication (WC) devices/antennas [mobile/“smart” phones and corresponding base antennas, cordless domestic (DECT: Digitally Enhanced Cordless Telecommunications) phones, “wireless fidelity” (Wi-Fi) routers for wireless Internet connection, “bluetooth” wireless connection among electronic devices etc.], are oscillating and highly variable at each moment, especially in their intensity. All types of WC EMFs consist of Microwave (MW) carrier waves (300 MHz-300 GHz), modulated, mainly by Extremely Low Frequency (ELF: 3–3,000 Hz) or Very Low Frequency (VLF: 3–30 kHz) signals, and included in on/off pulses repeated at various ELF rates. Moreover, they exhibit intense random variability in their signal amplitudes with frequencies in the Ultra Low Frequency (ULF: 0–3 Hz) band. The MW band is part of the wider Radio Frequency (RF: 300 kHz–300 GHz) band. Therefore, even though all WC EMFs are usually referred to simply as “RF” EMFs, in fact they are a combination of RF/MW, ELF and ULF EMFs (1–4). Figure 1A shows 2nd generation (2G) mobile telephony (MT) Global System for Mobile telecommunication (GSM) basic frame repetition, nominally 217 Hz, pulsations. Variability in both pulse amplitude and repetition frequency is evident as in all real-life WC signals. Newer systems 3G, 4G, 5G have basic frame repetition frequency (nominally) at 100 Hz and exhibit increasing variability in their pulsations/signals due to the increasingly higher amounts of variable information they carry (speech, images, video, Internet, etc.) (3). Figure 1B shows 100 and 200 Hz pulsations from a DECT phone.

These unique features make all anthropogenic, and most of all WC EMFs very different than the natural EMFs which only in specific cases are partially polarized and/or partially coherent to a small degree (3, 5). The geomagnetic field (GMF) and geoelectric field (GEF) are significantly polarized and coherent but static, with no significant variability. During magnetic storms, approximately every 11 years, there is a variability of about 20% in their normal intensities and then there are increased rates of disease and mortality in the human/animal populations (6).

It seems that the combination of polarization/coherence and low-frequency variability is the key to EMF-bioactivity. Polarized and coherent EMFs/EMR (in contrast to, e.g., light and other types of natural EMFs/EMR) possess net electric and magnetic fields, in addition to radiation intensity, which exert forces on every electrically charged/polar particle/molecule such as the mobile ions and the charged/polar macromolecules in all biological systems. It is those unique features that make all anthropogenic EMFs, and most of all WC EMFs, significantly more adversely bioactive than natural EMFs (3, 5, 7).

It has been repeatedly documented that modulated (especially in amplitude) or pulsed RF EMFs are significantly more bioactive than non-modulated or non-pulsing fields of the same carrier frequency and the same intensity with that of the pulses (8–29). [For reviews see (3, 30)]. In all cases, the reported effects were not accompanied by any significant heating of the exposed biological tissues, in other words they were “non-thermal.” This evidence implies that the non-thermal biological effects of WC EMFs are due to the included ELF pulsation/modulation.

In addition, ELF EMFs alone have been found independently to be bioactive, similarly to RF EMFs modulated or pulsed by ELFs, providing additional confirmation that actually the ELF pulsation and modulation EMFs are responsible for the non-thermal effects of WC EMFs and not the RF carrier EMFs (11, 18, 31–48). Again, in all cases, the described effects were non-thermal.

The evidence that the ELF/ULF and not the RF carriers of the anthropogenic/WC EMFs are those that induce the non-thermal effects, is in line with the fact that the physiological electrical activity in all forms of life is restricted to ULF/ELF EMFs. There is no physiological RF EMF in the living organisms, neither in the natural environment, despite the confusion and misinformation among the scientific community for the opposite (6, 49–52). The so called “cosmic microwaves” are actually infrared radiation reaching the Earth with a lower frequency due to the Doppler effect (50, 53). Thus, it is evident that the non-thermal biological and health effects attributed to “RF” EMFs are actually due to their included ELF pulsations, modulation, and variability. And there is practically no RF EMF in any technical application that is not combined with ELFs. All modern digital “RF” EMFs contain ELF pulsations, i.e., not only WC systems but also radars and radio/television broadcasting systems (3, 4, 7, 50, 54–56). Even though this is well documented it has escaped attention, and still authors look for different mechanisms for ELF and “RF” EMFs (57–59). Authors who report that they have found non-thermal EMF effects by non-modulated and non-pulsed RF carrier signals alone but do not provide the signal waveform [as, e.g., (60)], either are unaware of the existence of pulsations produced by almost every existing RF generator, or the effects they report are due to the onset and offset of the RF exposure (18, 56).

### 1.2 Anthropogenic ELF and WC EMFs: Biological and health effects

Multiple experimental findings associate exposure of laboratory animals or cells to man-made ELF or WC EMFs/EMR with Oxidative Stress (OS) due to Reactive Oxygen Species (ROS) overproduction, genetic damage/alterations (DNA damage, chromosome damage, mutations, etc.), cell senescence (cell aging and loss of replicative capacity), cell death, and related effects [see reviews in (7, 18, 43, 50, 61–66)].

More specifically, numerous *in vivo* or *in vitro* experimental-laboratory studies have shown genetic damage and related effects induced by man-made ELF or WC EMFs on a variety of organisms/cell types under various experimental conditions, especially in recent years. Representative such studies are (15, 16, 33–38, 42, 67–126). If we add studies that found induction of OS and/or cell senescence, the list becomes much longer (62–64, 127–133).

Several of these studies have found OS, and/or DNA damage with consequent cell death in reproductive cells of various animals, resulting in decreased reproduction or embryonic death. In particular, effects of WC EMFs on the DNA of reproductive cells reported by several studies on a variety of animals display a remarkable similarity (68, 71, 72, 74, 75, 97–99, 107, 115, 117, 125, 130). The genetic damage found in reproductive cells explains other findings that connect WC EMF exposures with insect, bird, and mammalian (including human) infertility (121, 134–144), miscarriages (145), or declines in bird and insect populations (especially bees) during the past 20 years (146–150). Significant decrease in reproduction (reduced egg laying, reduced development of reproductive cells, or embryonic death) after exposure to MT radiation, was identically observed in fruit flies (97, 98, 141, 142), chicken or quail embryos (71, 125, 151), bovine oocytes (137), birds (147, 148, 150), and bees (143). Similar effects are reported for amphibians (152, 153), rats and mice (107, 121, 135, 136, 138), and human sperm (decreased number and motility of spermatozoa) (134, 144). These remarkably similar findings in various animals and humans by different research groups can be explained by the cell death in reproductive cells or embryonic death after DNA damage observed in mouse or rat sperm cells (68, 107), fruit fly ovarian cells (72, 97–99), human sperm cells (74, 75), and quail embryos (71, 125).

It is again remarkable that the effects of purely ELF EMFs on reproductive cells and reproduction are very similar to those of WC EMFs (31, 33, 36–38, 42, 45, 46), further implicating ELF EMFs as a key bioactive agent.

Apart from the laboratory findings on genetic damage and infertility, epidemiological studies increasingly link man-made ELF or WC EMF exposures with health problems, genetic damage, and cancer in human populations. More specifically, ELF EMFs from power lines and high-voltage transformers (50–60 Hz) are linked to childhood leukemia and other cancer types (154–167) for magnetic field intensities down to 2 mG (0.2 μT) (159, 161), or distances from power lines up to 600 m (157), and electric field intensities down to 10 V/m (155). WC EMFs from various antennas, especially radio broadcasting and MT antennas, have been linked to various forms of cancer (168–171) and genetic damage (113, 172, 173). During the past 15–20 years epidemiological studies find an increasing association between mobile or cordless phone use and brain tumors in humans (174–185). For a review of EMF carcinogenicity studies see Yakymenko and Tsibulin (171).

Moreover, during the past 25 years, other epidemiological studies find association between exposure to MT/WC antennas/devices with reported symptoms of un-wellness referred to as “microwave syndrome,” or “electro-hypersensitivity” (EHS). The symptoms include headaches, fatigue, sleep disorders, and various other adverse effects (169, 186–196). A high percentage (~80%) of EHS self-reporting patients were found with increased OS in their peripheral blood (197). EHS symptoms have been reported to increase dramatically among people exposed to 5G WC antennas, and the ambient RF EMF levels in cities have also been found to increase significantly during the past 2 years, after the beginning of 5G rollout (198, 199).

Cancer in experimental animals after chronic exposure to MT/WC EMFs has also been reported (200, 201). A study of the US National Toxicology Program (NTP) found that exposure of rats to simulated 2G or 3G MT emissions (2 years, 9 h per day) induced brain cancer (glioma) and heart cancer (malignant schwannoma) for both lower and higher radiation levels than the officially accepted limits (202). The study also found significantly increased DNA damage (strand breaks) in the brains of exposed animals (124) confirming the tight link between DNA damage and carcinogenesis. An Italian life-span exposure study of rats to a simulated 2G MT EMF also found induction of heart schwannomas and brain glial tumors, confirming the results of the NTP study (203).

Other studies have reported no effects of ELF or RF/WC EMFs in all of the above end points [see reviews in (3, 18, 42, 43, 61–65, 141, 171, 204–213)], especially studies that employed simulated MT/WC exposures from generators with invariable parameters and no modulation. By contrast, more than 95% of the studies that employed real-life MT/WC exposures, from commercially available devices (mobile/cordless phones, Wi-Fi, etc.) with high signal variability, find effects (3, 7, 206, 209, 210, 214–216).

Regardless of real-life or simulated exposures, the majority of experimental studies (approximately 70%) either with “RF” (combined with ELF) or purely ELF EMFs do find effects (62–64, 206, 208). Jagetia (64) did an extensive review of laboratory studies addressing genotoxic effects of either ELF or RF/WC EMFs in a variety of biological systems, and found that among 207 studies, 144 (69.6%) found statistically significant genotoxic effects. The vast majority of reported effects were non-thermal, and the vast majority of employed EMFs contained ELF/ULF components.

The recorded human and animal carcinogenicity, the DNA/genetic damage, the OS findings, and the reproductive declines due to DNA damage in ovarian or sperm cells or embryonic death, all point toward the same direction: Man-made EMFs induce OS and DNA damage, infertility, cancer, and other related pathologies. The reason why the same effects are observed in a wide variety of animals such as mammals, birds, insects, etc., and humans, is that all biological and health effects initiate in cells and all cells are essentially identical in all animals, humans, and even plants. They have identical membranes, ions, ion channels and pumps, biomolecules such as DNA, RNA, proteins, etc., water, ROS, identical cellular organelles such as nuclei, mitochondria, ribosomes, endoplasmic reticulum, etc., and very similar metabolic processes and regulatory mechanisms. These similarities at the cellular level between all animals and humans are much more fundamental than differences in volume, mass, shape, macroscopic functions, intelligence, etc. As a result, any effect induced by EMFs in animal cells such as OS, DNA damage, etc., is expected to be induced also in the human cells, and vice-versa (7, 66).

The exposure levels in the vast majority of all the aforementioned studies were significantly below the officially accepted exposure limits for ELF and RF EMFs, which are recommended by a private organization called the International Commission on Non-Ionizing Radiation Protection (ICNIRP) to prevent discharges on humans in the case of ELF and acute heating of living tissues in the case of RF/WC EMFs. It is remarkable that this organization arbitrarily ignores the overwhelming evidence of non-thermal effects which constitute the vast majority of effects of anthropogenic EMFs, and yet, governments adopt its recommendations instead of following the Precautionary Principle which dictates the obvious, that no new technology should be applied unless those who promote it have proven its safety beyond any doubt (50, 217–226).

The International Agency for Research on Cancer (IARC) branch of the World Health Organization (WHO), has, since long time, classified both ELF and “RF” (in fact WC) EMFs as possibly carcinogenic to humans (Group 2B) (204, 205, 227). Based on additional scientific evidence after the 2011 IARC classification for “RF” EMFs, several studies have argued that “RF”/WC EMFs should be re-evaluated and classified as probably carcinogenic (Group 2A) or carcinogenic (Group 1) to humans (50, 63, 64, 66, 118–120, 171, 179, 182, 183, 218, 219). Moreover, studies have asked for the urgent application of the Precautionary Principle, stricter exposure limits, especially for WC EMFs, and a moratorium on 5G rollout (50, 215, 217, 218, 225, 228).

### 1.3 Ionization in living tissue by “non-ionizing” EMFs

As indicated by the long list of laboratory and epidemiological studies, man-made EMF exposures are linked to OS, genetic damage, infertility, EHS, and cancer. Damage to DNA or other biological molecules involves breakage of chemical bonds and chemical alterations, in other words ionization. Man-made EMFs with frequencies up to the low limit of infrared (0–3 × 10^11^ Hz) examined here cannot directly break chemical bonds and cause ionization, except for very strong field intensities (≥10^6^ V/m) (229, 230). Such field intensities are rarely present in the environment, apart from very close proximity to high-voltage power lines and transformers, or very close to atmospheric discharges (lightning). How are then man-made EMFs at environmental intensities capable of ionizing DNA and other biological molecules? What is the unique property that makes man-made EMFs capable of inducing adverse biological/health effects in contrast to natural EMFs including light? It has been shown that this unique property is polarization and coherence combined with low frequency (ULF/ELF) variability (2, 3, 5, 7).

In the present work we provide an updated description of how man-made EMFs at non-thermal levels are capable of inducing dysfunction of Voltage-Gated Ion Channels (VGICs) in cell membranes, triggering ROS overproduction and OS, which in turn is responsible for most, if not all, known adverse biological/health effects including DNA damage and related pathologies. Thus, ionization of biological molecules occurs indirectly after man-made EMF exposure, mediated by the produced ROS in the cells (2, 210).

## 2 Anthropogenic ELF or WC EMFs and OS: Experimental evidence

Yakymenko et al. (62) reviewed 100 published experimental studies that examined OS in living cells from a wide variety of organisms (humans, rats, mice, rabbits, quail embryos, plants etc.) exposed *in vitro* or *in vivo* to RF/WC EMFs. From those studies, 93 found increased OS expressed as activation of key pathways generating ROS overproduction, peroxidation, oxidative damage of DNA, changes in activity of antioxidant enzymes, etc. In a more recent update, Yakymenko and Tsibulin (63) found that among 131 published peer-reviewed studies looking for oxidative effects of RF/WC EMFs at non-thermal intensities, in most cases pulsed/modulated by ELF EMFs, 124 (95%) confirmed statistically significant oxidative effects on various types of biological systems. And among 39 published studies on oxidative effects of purely ELF EMFs, 36 of them (92%) also found significant oxidative effects induced by the exposure. Therefore, it is well-documented that anthropogenic EMF exposures cause ROS overproduction and OS in living cells, which in turn is responsible for the observed DNA damage, infertility, cancer, and other related pathologies.

Even though ROS at sub-toxic levels in the cells act as signaling molecules involved in various physiological cellular processes, they can also damage biological molecules (such as lipids, proteins, and nucleic acids) causing various diseases when they are in excess (231–234). Most ROS are free radicals. Free radicals are extremely unstable and reactive molecules containing an unpaired electron denoted by a dot (•) in their chemical formula. They possess a very strong tendency to chemically react with other molecules and/or with each other in order to couple their unpaired electron, balance electron spins, and become stable. This extreme reactivity is the reason why they have extremely short lifetimes. Most ROS react rapidly with surrounding biomolecules, causing chemical alterations (235, 236).

Two important initial free radical ROS found in cells after exposure to man-made EMFs are the superoxide anion (O_2_•^−^) and the nitric oxide (NO•) (62, 63, 125). The superoxide anion free radical may be converted into hydroxyl radical (OH•), or react with nitric oxide to form peroxynitrite (ONOO^−^). Both products (especially the hydroxyl radical) are very reactive ROS with biological molecules, especially DNA (62, 125, 237, 238).

## 3 Biochemistry of ROS

### 3.1 ROS sources in the cells: Identity and function: Dependence on ion concentrations

#### 3.1.1 Mitochondria

A major source of ROS in all cells is the Electron Transport Chain (ETC) in the inner membrane of the mitochondria (62, 65, 234, 235, 239), likely contributing 50–80%, or even 90% of total cellular ROS production under normal conditions (240). Electron leakage from Complexes I and III of the ETC is a significant source of ROS, especially superoxide anion free radical (O_2_•^−^) after adherence to molecular oxygen (241):


(1) $$O_{2}+e^{-}\to O_{2}{•}^{-}$$

Certain cell types such as neurons or spermatozoa have high energy demands and thus high mitochondrial activity, making them particularly susceptible to OS from mitochondrial ROS production (65, 242).

The large amounts of energy (in the form of Adenosine Triphosphate-ATP) required for the maintenance of a cell’s aerobic life are generated predominantly by oxidative phosphorylation in the mitochondria at the expense of molecular oxygen, which is reduced to water by 2 electrons per water molecule terminating in complex IV of the ETC [2H^+^ + 1/2O_2_ + 2e^−^ → H_2_O] (65). However, a fraction of the electrons will ‘leak’ from the ETC and partially reduce oxygen (Equation 1). The result is that a small amount (~2%) of oxygen during this process is converted to superoxide anion radical, hydrogen peroxide and related ROS. Cells and tissues reliant on oxidative phosphorylation have evolved effective antioxidant measures to keep these modest ROS levels in check and inhibit OS. Nevertheless, perturbation of electron flow and/or the homeostasis of the mitochondrial environment can dramatically elevate the risk of OS. This is exemplified in spermatozoa, where minimal cytoplasm and abundant substrates for oxidative chemistry offer a platform for run-away levels of ROS with minimal perturbation of their mitochondrial environment (65, 243). Electron leakage from the ETC in spermatozoa under WC EMF exposure was recorded to be sourced from complex III, as this was tested in parallel with the use of complex III-inhibitors (115). See Miller et al. (65) for a more detailed view on mitochondrial ETC and ROS production.

When considering the mitochondrial origins of OS, Ca^2+^ plays a key role. Physiological increases in mitochondrial Ca^2+^ can stimulate ATP production when energy demands are high, however this can also result in elevated ROS generation. Excessive Ca^2+^ accumulation can lead to mitochondrial dysfunction and a drop of ATP production, but importantly, further elevates ROS production and apoptotic factors (244). Ca^2+^ can act upon the mitochondria through its regulatory activity on key mitochondrial specific dehydrogenases, such as pyruvate dehydrogenase, which is an intrinsic mediator of electron flow through the ETC and influences ROS production (245). Therefore, careful control of Ca^2+^ levels in the mitochondria is a key factor in ROS homestasis, with any dysfunction of Ca^2+^ channels induced by EMFs potentially leading to ROS overproduction.

Voltage-gated anion channels in the outer membrane of the mitochondria regulate Ca^2+^ entry into the intermembrane space and the matrix. While increased levels of Ca^2+^ in the mitochondria stimulate O_2_•^−^ synthesis by the ETC, the presence of the other “initial” ROS, NO•, inhibits complex IV of the ETC causing additional electron leakage and increased O_2_•^−^ production (235, 244). Thus, the two important primary ROS (NO• and O_2_•^−^) act in synergy in the mitochondria, and increased levels of NO• stimulate further production of O_2_•^−^.

ROS overproduction in the mitochondria may damage DNA both in the mitochondria and the nucleus, and may initiate a signaling cascade leading to apoptosis. In turn, excessive apoptosis induced by increased ROS levels, has been linked to inflammatory diseases and cancer (246).

#### 3.1.2 NADPH/NADH oxidases (NOXs)

These plasma membrane enzymes, found in abundance in all cells, normally generate ROS for the elimination of invading microorganisms. NAD(P)H oxidase is an enzyme exhibiting different affinities for its two substrates; Nicotinamide Adenine Dinucleotide Phosphate (NADPH), and Nicotinamide Adenine Dinucleotide (NADH), with the NADPH substrate being the most common (247–251). The NOXs catalyze the production of superoxide anion free radical by transferring electrons to oxygen from NAD(P)H according to the reaction:


(2) $$\mathrm{NAD}\left(P\right)H+{\mathrm{2O}}_{2}\to \mathrm{NAD}{\left(P\right)}^{+}+{\mathrm{2O}}_{2}{•}^{-}+H^{+}$$

Thus, they also generate an ETC for the reduction of extracellular O_2_ to O_2_•^−^. The activity of NOX is intimately connected with H^+^ channels and the enzyme may even act as a H^+^ VGIC itself, due to its gp91^phox^ transmembrane subunit (252–254).

NOXs are also activated by cytosolic Ca^2+^ and possess a Ca^2+^-binding site, apart from their H^+^ voltage-gated channel (gp91^phox^ domain) (251). Thus, perturbation of intracellular concentrations of either H^+^ or Ca^2+^, after irregular gating of their VGICs, can affect NOX function and trigger ROS (over)production.

NOXs may contribute 10–30% of total ROS production in neurons under basal conditions. However, their contribution can increase significantly during neuronal activation or inflammation. NOXs, particularly NOX2 and NOX4, are expressed in neurons and play roles in synaptic plasticity, neuronal signaling, and neuroinflammation. Their activity can be upregulated in response to various stimuli, leading to increased ROS production (255).

Some NOX isoforms, such as NOX2 in phagocytes, are directly activated by Ca^2+^ through interactions with regulatory subunits like p47phox and p67phox, and increased intracellular Ca^2+^ levels can promote ROS production. Ca^2+^ can also indirectly regulate NOX activity through various signaling pathways, such as those involving protein kinase C and other kinases (256).

The NOXs have been identified as a key target for man-made EMFs. Friedman et al. (257) found rapid ROS production by the NADH oxidase in cultured cells after a few min exposure to simulated mobile phone EMF.

A common function in the NOXs and the mitochondria is the generated ETC. In both cases, the primary ROS produced is superoxide anion radical (O_2_•^−^), which may finally convert to hydroxyl radical (OH•), or peroxynitrite (ONOO^−^) as shown below (Equations 3–7).

#### 3.1.3 Nitric oxide synthases (NOS)

These are specific enzymes found in various locations in all animal and plant cells, and as denoted by their name, produce nitric oxide free radicals (NO•). Several NOS enzymes have been well described, such as the neuronal NOS (nNOS), or the endothelial NOS (eNOS) which are plasma membrane enzymes. Their activation seems to be dependent on intracellular calcium levels and calmodulin. Increased levels of intracellular Ca^2+^ stimulate NO• synthesis by the NOS. NO• is ubiquitous in cells and tissues in all vertebrates as an intercellular messenger and certain studies suggest that it is not particularly toxic by itself, but it may easily be converted to peroxynitrite which is particularly toxic (237, 258). Increases in Ca^2+^ and NO• levels in cells have been found to be triggered very rapidly (within a few seconds) by EMF exposure (259), with the induction of DNA damage by peroxynitrite blocked by NOS inhibitors (260) and antioxidants (238, 261, 262). While nNOS and eNOS, are Ca^2+^-dependent enzymes activated by increased intracellular Ca^2+^ levels, and promoting NO• production, under certain conditions NOS can become “uncoupled” due to substrate depletion or cofactor deficiency, triggering the cell machinery to produce O_2_•^−^ instead of NO• by other ROS sourses/enzymes (263).

#### 3.1.4 Xanthine oxidase (XO)

Xanthine Oxidase (XO) is another important source of ROS in the cytoplasm of living cells. XO catalyzes the oxidation of hypoxanthine to xanthine and then to uric acid, producing superoxide anion radical (O_2_•^−^) and hydrogen peroxide (H_2_O_2_) as byproducts. XO is involved in purine metabolism and can be a significant source of ROS under certain conditions, such as ischemia–reperfusion injury (264).

#### 3.1.5 Other ROS-generating/promoting enzymes

Such enzymes in cells are, e.g., cytochrome P450 (CYP), lipoxygenases, cyclooxygenases, myeloperoxidases and others (240). They may contribute to ROS generation differently under different physiological conditions. For example, CYP is localized in the endoplasmic reticulum and is crucial for the metabolism of various endogenous and exogenous compounds, including drugs, steroids, and fatty acids. CYP reactions can generate ROS as byproducts, particularly during the catalytic cycle when oxygen is activated (265).

The ionic balance within a cell (also called electrochemical balance) is most crucial for ROS production, and both ion channels and pumps can trigger ROS production by the above described ROS sources. The ion pumps (active ion transporters) such as the Na^+^/K^+^ pump (ATPase), in coordination with the ion channels (passive ion transporters) determine the membrane voltage, the electrochemical balance, the cell’s homeostasis, the redox status (concentrations of reducing and oxidizing molecules), and the cell’s volume among other functions. Ca^2+^ and K^+^ channels are involved in cell proliferation, apoptosis, and carcinogenesis (266, 267). In addition, they are both involved in iron entry into the cells (268–270). Iron catalyzes the production of OH• via the Fenton reaction and thus, impaired function of these channels can promote cellular toxicity. Dysfunction of Na^+^, K^+^, Mg^2+^, and Ca^2+^ VGICs will affect the function of the Na^+^/K^+^ pump (ATPase), and Ca^2+^ pumps in the plasma membranes of all cells.

In addition to its role as an ion pump, Na^+^/K^+^-ATPase operates as a signal regulator, transducing signals from the plasma membrane to the intracellular organelles and acting as a normalizer of the Na^+^/K^+^ balance in cells after, e.g., VGIC dysfunction (231, 234, 271). It has long been shown that the activity of the Na^+^/K^+^-ATPase is affected by ELF EMFs (44, 272, 273), and that changes in its activity are linked to ROS production by the mitochondria and in turn, increased mitochondrial ROS production stimulates the signaling function of the enzyme forming a positive amplification feedback loop (274, 275). Thus, changes in the activity of the Na^+^/K^+^-ATPase due to EMF-induced dysfunction of VGICs can stimulate ROS production by the mitochondria and the process can be amplified by increasing ROS levels (2, 210).

### 3.2 ROS action on DNA and other biological molecules

DNA damage induced by OS/ROS leading to mutations and disease has been well documented since long time. The effects of ROS on DNA commonly result in altering the nucleotide bases which directly affects their paring elevating mutational load, altering a sugar (deoxyribose), breaking a covalent bond between deoxyribose and nucleotide base, and causing single- and double-strand breaks, further increasing the repair burden on the affected cells and tissues (2, 210, 243, 276–279).

We have described the evidence regarding the generation of primary ROS, NO• and O_2_•^−^, by the ROS sources in the cells after anthropogenic ELF or WC EMF exposure (section 2). These initial ROS are converted to other even more potent ROS, hydroxyl radical and peroxynitrite, which we call “final” ROS, and are those that mainly damage DNA and other biological molecules.

#### 3.2.1 Peroxynitrite

Increased concentrations of NO• and O_2_•^−^ within a cell lead to peroxynitrite (ONOO^−^) overproduction after reaction among the two initial ROS, as follows:


(3) $$\mathrm{NO}•+O_{2}{•}^{-}\to {\mathrm{ONOO}}^{-}$$

Peroxynitrite is a strong non-free radical ROS which can damage critical molecules including DNA (280). Both nitric oxide and peroxynitrite can diffuse anywhere within the cell and, thus, act directly on DNA or other molecules. The effects of peroxynitrite on DNA include base and sugar oxidative modifications, and DNA single-strand breaks (258, 281). Of the four DNA bases, guanine is the most vulnerable to peroxynitrite (282). DNA single-strand breaks caused by peroxynitrite is a well-documented effect which can be prevented by use of calcium channel blockers and antioxidants (238, 258, 261, 262, 283). Pall (238) noted a connection between EMF-induced dysfunction of Voltage-Gated Calcium Channels (VGCCs) and NO•/ONOO^−^ overproduction. Peroxynitrite can also decompose easily in the presence of H^+^ to form OH• and NO_2_• (284, 285):


(4) $${\mathrm{ONOO}}^{-}+H^{+}\to \mathrm{OH}•+{\mathrm{NO}}_{2}•$$

#### 3.2.2 Hydroxyl radical

The superoxide anion radical (O_2_•^−^) produced by the mitochondria or the NOXs, is catalyzed by superoxide dismutase (*SOD*) in the cytosol or the mitochondria converting to hydrogen peroxide (H_2_O_2_) (248, 280, 286):


(5) $${\mathrm{2O}}_{2}{•}^{-}+{\mathrm{2H}}^{+}\overset{SOD}{\to }\;H_{2}O_{2}+O_{2}$$

H_2_O_2_ can move to any cellular site, including the nucleus, where it can be converted to the most potent hydroxyl radical (OH•) which can damage any biological molecule, including DNA (287–291).

OH• is considered the most potent oxidant of DNA. It is mainly produced by iron-catalyzed conversion of H_2_O_2_ via the Fenton reaction (292): Fe^2+^ is oxidized by H_2_O_2_ to Fe^3+^, producing a OH• radical and a hydroxide ion (OH^−^). Fe^3+^ is then reduced back to Fe^2+^ by another H_2_O_2_ molecule, producing a hydroperoxyl radical (HOO•) and a proton. The net effect is the conversion of two hydrogen peroxide molecules to produce OH• and HOO•, with water (H^+^ + OH^−^) as a byproduct (2, 210):


(6) $$2\;H_{2}O_{2}\to \mathrm{OH}•+\mathrm{HOO}•+H_{2}O$$

Another way for OH• production is the Haber-Weiss reaction (293):


(7) $$O_{2}{•}^{-}+H_{2}O_{2}\to O_{2}+\mathrm{OH}•+{\mathrm{OH}}^{-}$$

The OH• radical can break biological macromolecules (R-R or R-H) in its immediate environment or subtract atoms from them (such as the various hydrogen atoms of the deoxyribose molecule) by breakage of covalent bonds. This results in chemical alterations in the macromolecules and production of new free radicals such as R• or RO• (2, 210, 294, 295).


(8) R−R+OH•→ROH+R•

(9) $$RH+OH•\to R•+H_{2}O$$

(10) $$RH+OH•\to \mathrm{RO}•+H_{2}$$

The new free radicals will further react with other molecules and with each other resulting in additional chemical alterations.

A more specific example is the action of OH• on DNA that results in the breakage of the DNA chain (single- or double-strand breaks). The backbone of each strand of the DNA is formed by phosphodiester bonds between two successive deoxyribose (DOX) molecules and a phosphate (–PO_4_–) between them. For a strand breakage, the phosphodiester bond needs to break. The double-strand breaks (breakage of both strands of the double helix at the same point) are the most severe and in most cases irreparable damages that lead to DNA fragmentation, mutations, cell death etc. The breakage of the phosphodiester bond by two hydroxyl radicals can be written as:


(11) $$\begin{array}{l} \left[-\left(b\right){\mathrm{DOX}}_{i\left(3΄\right)}–{\mathrm{PO}}_{4}{–}_{\left(5΄\right)}{\mathrm{DOX}}_{i+1}\left(b\right)–\right]+\mathrm{2OH}•\to \\ \left[-\left(b\right){\mathrm{DOX}}_{i\left(3΄\right)}–\mathrm{OH}\right]+{\mathrm{PO}}_{3}+\left[\mathrm{HO}{–}_{\left(5΄\right)}{\mathrm{DOX}}_{i+1}\left(O-b\right)–\right] \end{array}$$

By (b) we denote the DNA bases connected to the DOX_i_ and DOX_i + 1_ molecules. (3΄) and (5΄) are the carbon atoms in successive deoxyribose molecules that form the phosphodiester bond with the phosphate. In the products of the reaction, PO_3_ is separated from the DNA molecule and is not anymore part of the phosphodiester bond that kept the strand intact. The second base is oxidized by the hydroxyl radical as well (to form O-b) in addition to the breakage of the strand (296). The breakage forms OH at the terminals of the broken strands. The TUNEL (Terminal deoxynucleotide transferase dUTP Nick End Labeling) assay used in biology to detect fragmented DNA, specifically detects the free OH terminals of the broken strands (42, 97, 98).

## 4 Anthropogenic EMFs and VGICs

### 4.1 VGICs: Most sensitive electromagnetic sensors in living organisms

Previous studies have hypothesized the existence of specific electro/magneto-sensor organs/cells in animals/humans in order to explain the biological effects of EMFs [see review in (297)]. This is not necessary, as all cells in all animals including humans and even plants are equipped with the most sensitive EMF-sensors which are no other than the VGICs, the most abundant class of ion channels in all cell membranes (231, 234, 271, 297, 298).

**Figure 2:**
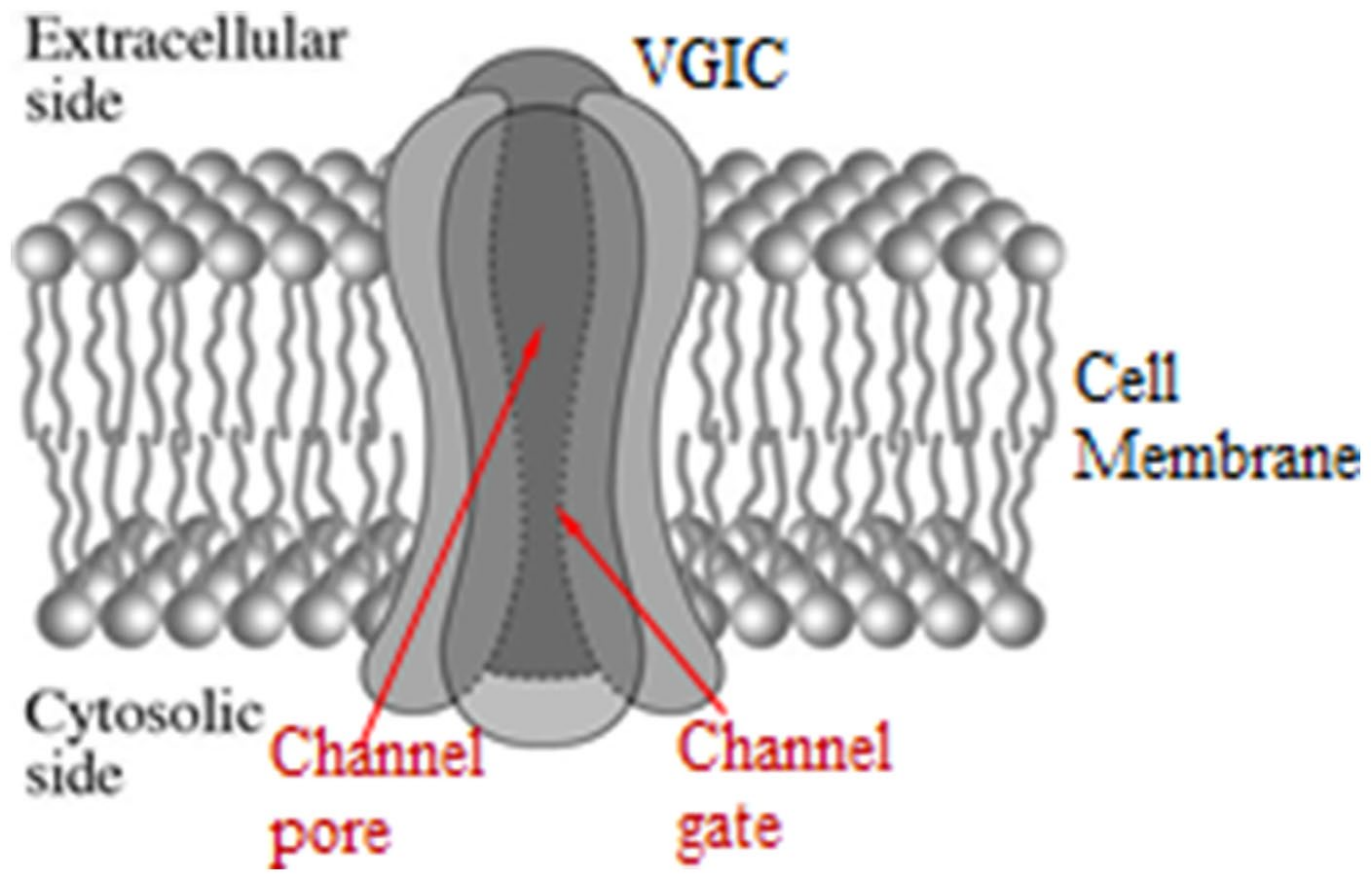
Schematic representation of a VGIC with its four main units, the pore, and the gate in opened state. Each main unit consists of six parallel *α*-helices (S1–S6). The voltage-sensors of the VGIC are the four S4 helices, one in each main unit, in symmetrical positions around the pore [adapted from Panagopoulos et al. (297)].

Normally VGICs convert between open and closed states by membrane voltage changes *dV* ≥ 30 mV which exert forces on their voltage-sensors. More specifically VGICs respond to changes between ⁓30 and ⁓100 mV. The voltage sensors of the VGICs are four symmetrically arranged, transmembrane, positively charged parallel *α*-helices (subunits), each one named S4. They occupy the 4th position in a group of 6 parallel α-helices (S1-S6), and are the closest helices to the pore apart from the S5-S6 helices which form the pore walls. The channel consists of four identical such groups (main units I-IV) in symmetrical positions around the pore of the channel (Figure 2). The sensors are positive Lys and Arg amino-acids in the S4 helices. The effective (net) charge on each S4 has been calculated to be *q =* 1.7*q_e_*, where *q_e_* is the elementary charge. The positive charges of the S4 sensors are paired with negative charges from adjacent helices so that the net charge on the walls of the pore is zero. The ions pass dehydrated and in single file through the channel gate, the narrowest part of the pore (Figure 2). At least four dehydrated mobile ions are very close to the S4 sensors at a distance of less than 1 nm, as, except for the ion(s) that may be passing through the gate any moment or is just outside the gate ready to pass, at least three more are bound in specific ion-binding sites very close to the gate (234, 297, 299–304).

### 4.2 Anthropogenic EMFs and VGIC dysfunction: The IFO-VGIC mechanism

A biophysical mechanism for EMF-induced biological effects has been described in Panagopoulos et al. (2, 5, 297, 305, 306), and Panagopoulos (302). It explains, in standard physics and biology, how polarized, coherent and slow-varying (ULF/ELF/VLF) EMFs, even at very low field intensities, can cause irregular gating (opening/closing) of VGICs in cell membranes with consequent disruption of the cell’s electrochemical balance, redox state and homeostasis. As ELF/ULF/VLF EMFs are basic components of the WC EMFs, this mechanism accounts for the biological effects of the vast majority of all man-made (polarized, coherent, and varying) EMFs.

**Figure 3:**
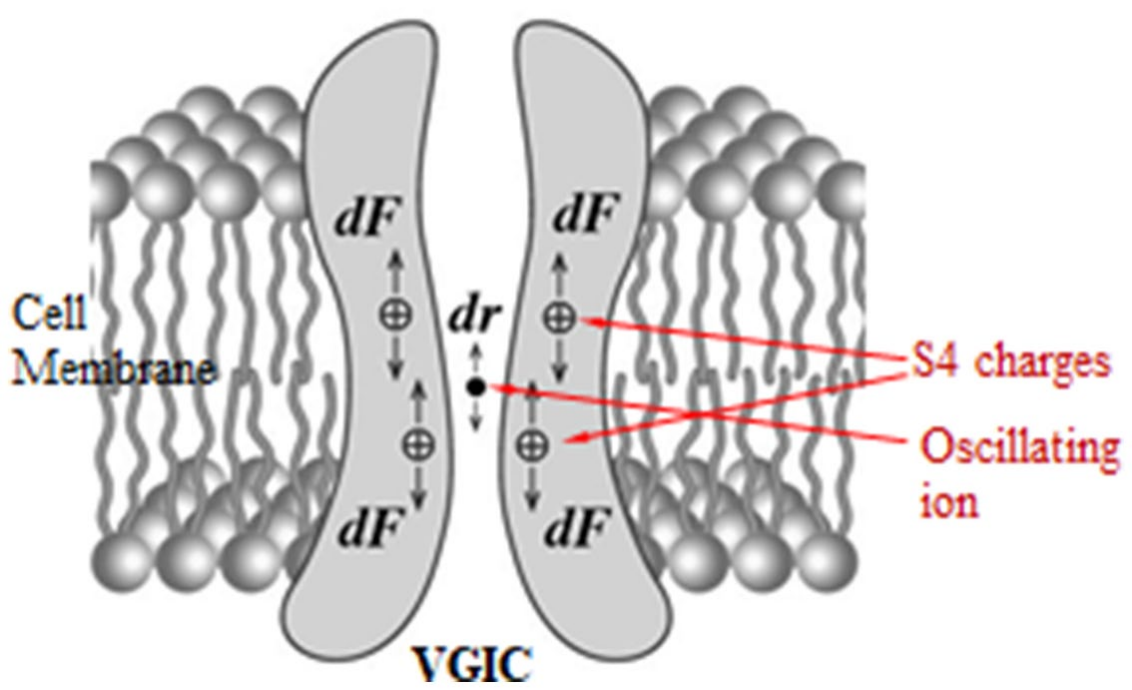
Forces *dF* exerted on the S4 positive charges in a VGIC due to the displacement *dr* of an oscillating ion in the channel pore [adapted from Panagopoulos et al. (297)].

While VGICs are normally gated by ⁓30–100 mV voltage changes in the very strong transmembrane field, in other words respond to field changes between 3 × 10^6^ and 10^7^ V/m, they may also respond to very weak polarized, coherent, and slow-varying EMFs down to ⁓10^−5^ V/m via the forced-oscillation such EMFs induce on mobile ions in close proximity (<1 nm) to the sensors (ion forced-oscillation: IFO). This occurs because the force exerted on the S4 sensors by oscillating ions in close proximity, depending upon the inverse third power of the distance between charges (see Equation 12 below), is much greater than a direct force from an externally applied EMF which depends upon the first power of the applied field (2, 5, 297). The aforementioned (at least) four ions close to the pore gate, once forced to oscillate in parallel and in phase, exert constructive coordinated forces on the S4 sensors able to gate the channel (Figure 3).

Forces exerted by an external polarized EMF on any mobile ion within any VGIC, cause a displacement *dr* of the ion from its “initial” position which in turn exerts an additional Coulomb force *dF* on the S4 voltage-sensors of the VGIC which can result in the opening/closing (gating) of the channel (2, 5, 297, 305, 306) (Figure 3). This additional Coulomb force on each S4 sensor due to the ion displacement *dr* is given by the equation:


(12) $$dF=-\frac{q\cdot zq_{e}}{2\pi \varepsilon \varepsilon_{o}r^{3}}dr$$

with *q =* 1.7 *q_e_* the effective (net) charge of the S4 sensor, z*q_e_* the mobile ion charge with *z* the ion valence (e.g., *z* = 1 for K^+^, Na^+^ or *z* = 2 for Ca^2+^ ions), *q_e_* = 1.6 × 10^−19^C the elementary charge, *ε_o_ =* 8.854 × 10^−12^ N^−1^m^−2^C^2^ the vacuum permittivity, *ε* ⁓ 4 the relative permittivity of the ion channel, and *r* = 1 nm the “initial” distance between the two charges (2, 299, 305–308).

In the simplest case of a harmonically oscillating applied EMF, the maximal ion displacement in one direction during its oscillation is 2*A* (*A* the amplitude of the forced oscillation). By solving the corresponding differential equation [see (302, 305)] we get that for an applied electric field,


(13) $$A=\frac{E_{o}zq_{e}}{\beta \omega }$$

where *E_o_* is the intensity amplitude (maximal value) of the applied field, *ω* = 2*πν* (*ν* the frequency of the applied field), and *β* the damping coefficient during the ion oscillation (found to be within channels $\beta =\frac{E_{m}zq_{e}}{u_{o}}$ ≅ 6.4*z* × 10^−12^kg/s, with *E_m_* ~ 10^7^ V/m the transmembrane electric field, and *u_o_* = 0.25 m/s the ion maximal velocity through an open channel).

When the VGIC is gated physiologically by membrane voltage changes *dV* ≥ 30 mV, the minimum force on each voltage sensor that causes gating is calculated to be *dF* = 8.16 × 10^−13^ N, which, according to Equation 12, corresponds to a minimum coordinated displacement *dr* of four *z*-valence ions within the channel at ~1 nm distance from the sensors (2, 297), *dr* = 10^−12^/*z* (in m), and thus in order for an applied electric field to be able to gate the VGIC, the max ion displacement (2*A*) must satisfy the condition:


(14) $$2\frac{E_{o}zq_{e}}{\beta \omega }\geq 10^{-12}/z$$

For double-valence cations (*z* = 2) (e.g., Ca^+2^), this condition finally becomes:


(15) $${Ε}_{o}\geq 0.6\;\nu \times 10^{-4}\;\;\;\left(\nu \;\mathrm{in}\;\mathrm{Hz,}{Ε}_{o}\;\mathrm{in}\;\mathrm{V/m}\right)$$

**Figure 4:**
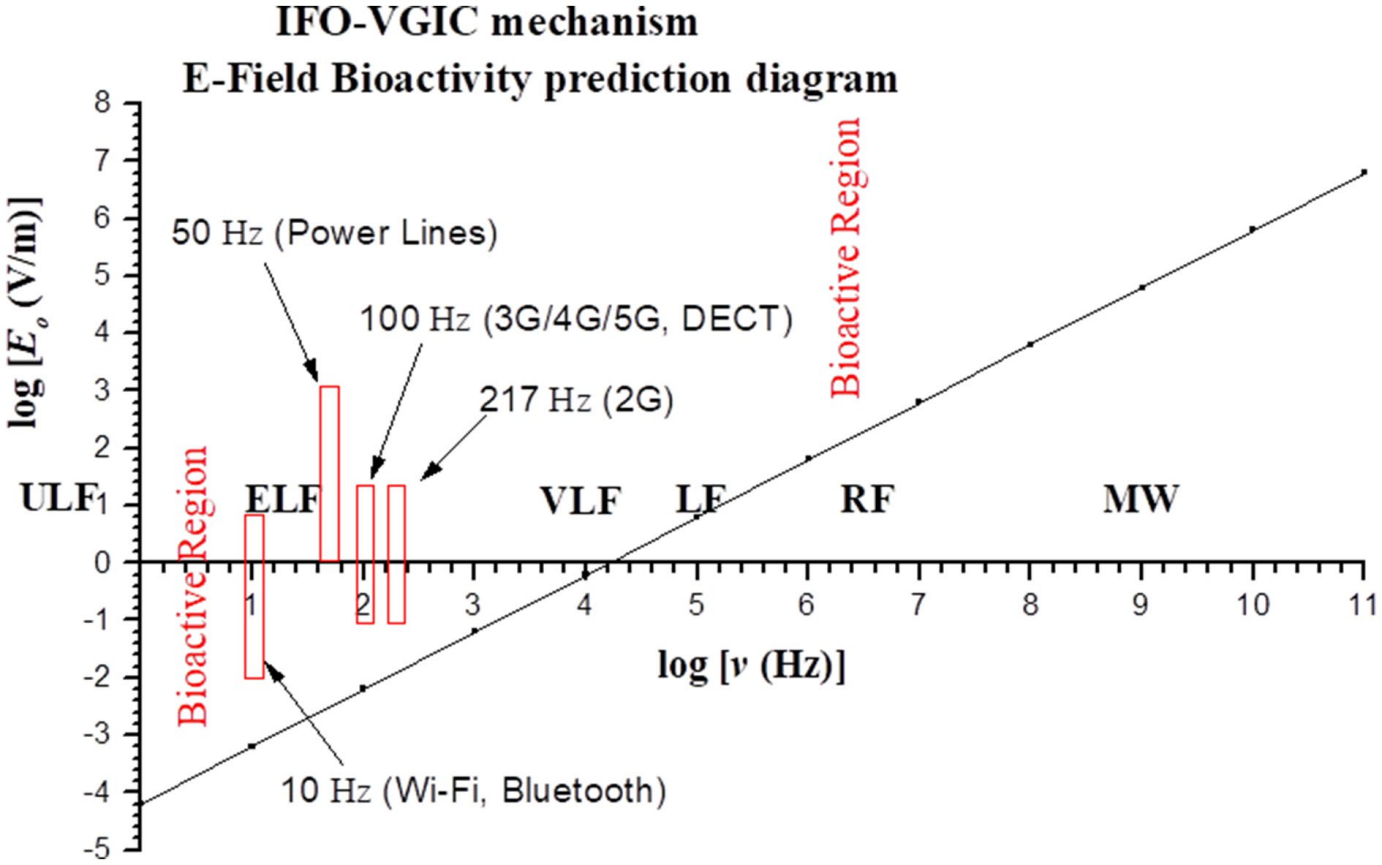
Graph (in logarithmic scales) showing the bioactive combinations of EMF intensity and frequency (above line) as predicted by the IFO-VGIC mechanism. The intensity-frequency combinations of all known anthropogenic EMF sources linked to adverse bioeffects are within the predicted bioactive region (2, 302).

This is the condition for electric fields in order to be bioactive with respect to field frequencies, and is represented in logarithmic scales (log*Ε_o_*, log*ν*) by the area above the line (including the line) in Figure 4 (“bioactive region”). The intensity-frequency combinations of all known anthropogenic EMF sources linked to adverse biological/health effects are within the predicted bioactive region. As the frequency of the applied EMF increases from ULF/ELF to VLF/LF (Low Frequency: 30–300 kHz) the required minimum field intensity in order to be able to induce effects increases considerably, and purely RF/MW EMFs need to have very high intensities (hundreds or thousands of V/m) in order to be bioactive.

The maximal ion displacement (2*A*) expresses the potential of the applied EMF to cause VGIC gating and initiate biological effects, in other words it represents the biological activity (or bioactivity) of the applied EMF:


(16) $$EMF\;bioactivity=2\frac{E_{o}zq_{e}}{\beta \omega }$$

Thus, the IFO-VGIC mechanism finds that the biological activity of an EMF is proportional to its maximum intensity and inversely proportional to its frequency, meaning that the reported effects in the literature are induced by low-frequency (ULF/ELF/VLF), and not high frequency (purely RF/MW), EMFs. Moreover, it finds that pulsing EMFs are significantly more bioactive than corresponding continuous-wave (non-pulsing) EMFs (see analysis in (2, 297, 302)).

According to the IFO-VGIC mechanism, VGICs respond to changes of polarized, coherent and slow-varying electric fields down to ⁓10^−5^–10^−4^ V/m, which is in impressive agreement with the threshold intensities of ELF man-made EMFs reported to induce biological effects in cell/tissue cultures (309, 310), and be sensed by electrosensitive animals (311, 312).

Apart from the electric fields, in fast moving animals/humans, the magnetic fields become increasingly bioactive with increasing velocities, and the same mechanism has explained animal orientation and navigation in the GMF (297).

It is known that living organisms are not particularly affected by static electric or magnetic fields but mostly by oscillating/varying (and polarized) ones. This is consistent with the IFO-VGIC mechanism, as VGICs are not gated by the normal static voltage/electric field across the cell membrane, but only by membrane voltage changes of the order of 30% in this voltage/field that cause membrane depolarization. In other words, VGICs do not respond simply to the presence of an invariable (static) electric field, otherwise they would open/close chaotically all the time and no life could be maintained. The same holds for the static magnetic fields, which can become bioactive with a variable animal velocity (297). This is the reason why the GMF and the GEF are not particularly bioactive under normal conditions but they become bioactive when ⁓20% changes in their normal intensities occur during magnetic storms (3, 6, 7, 297).

Thus, the IFO-VGIC mechanism predicts that polarized/coherent and slow-varying EMFs cause VGIC dysfunction (irregular gating), and this is today verified by many experimental studies [see (313–317), and reviews (44, 210, 238, 298, 318)].

## 5 VGIC dysfunction leading to OS: Connecting the dots for a comprehensive mechanism of EMF-induced biological and health effects

How can the initial ROS (O_2_•^−^ and NO•) generated after EMF exposure be produced by VGIC dysfunction? This was a missing link until recently when we specifically looked for such evidence (2, 210). We realized that even though plenty of data connecting impaired ion channel function and induction of cell death or cancer had been available for a long time (266, 267), and even though most ion channels, especially cation channels, are VGICs, the connection between VGIC dysfunction induced by EMF exposure and OS (2, 210, 238, 319–322) leading to DNA/cellular damage, had escaped the necessary attention.

Many studies have found a connection between Ca^2+^, K^+^, Na^+^, and Cl^−^ VGIC dysfunction with OS and related pathologies (238, 319, 321, 322). It is repeatedly shown that VGIC dysfunction induced by anthropogenic EMFs can trigger immediate ROS generation in the cells with this effect significantly diminished by the use of specific ion channel blockers (238, 259, 314, 316). Recent research further confirms the connection between VGIC dysfunction and ROS (over)production. For example, ROS overproduction through the activation of NADPH oxidase by extracellular tau-protein in co-cultures of neurons and astrocytes was reduced in the presence of nifedipine, inhibitor of Ca^2+^ VGIC (323). Epithelial cell death associated with elevation in ROS was prevented by lidocaine, a well-known Na^+^ VGIC inhibitor with antioxidant effects (324). Induced ROS production in murine microglia was inhibited in a dose-dependent manner by K^+^ VGIC blockage, and to a more limited degree, by Cl^−^ channel blockage (325).

Various pathological conditions, including neurodegenerative diseases, termed “channelopathies”, were discovered to be caused by ion channel dysfunction. Impairment of either voltage- or ligand-gated ion channels has been identified as a cause of neurological diseases. The ion channels involved include Ca^2+^, K^+^, and Na^+^ VGICs (318, 326). Multiple studies have documented the connection between Ca^2+^, K^+^, Na^+^, and Cl^−^ channel dysfunction and the development of OS-related pathologies (321). Ion channel dysfunction leading to OS is also a common cause of degenerative Central Nervous System (CNS) diseases of various genetic etiologies, and is a common factor in neurological disorders. The role of ion channels in neurodegenerative disorders associated with OS has now been recognized, as the ion channels undergo functional adjustments in such conditions (318, 327).

It is evident that the function of ion pumps and channels controls the intracellular concentrations of mobile ions, and in turn the function of the cellular systems/enzymes that produce ROS (2, 210). Any dysfunction in ion channels will affect the otherwise carefully controlled intracellular ionic concentrations, disrupting the cell’s electrochemical balance and homeostasis, including the intracellular redox status which is an index of the ROS content in the cell. From the evidence highlighted here, it follows that disturbance of ion homeostasis can trigger OS by ROS overproduction and subsequent DNA damage. Inversely, the intracellular redox status can alter the gating properties of ion channels and trigger opening or closing of Ca^2+^, Na^+^, and K^+^ channels in order to reinstate homeostasis (318, 331). Ion channels are thus gate keepers of redox status, and the cell’s electrochemical balance and homeostasis (305, 328).

For example, Ca^2+^ is a critical signaling factor, regulating many cell functions including cell proliferation, differentiation, and apoptosis (233, 244, 267, 329). Alterations in intracellular Ca^2+^ levels are decoded by Ca^2+^-sensors, which initiate signaling for various physiological processes (330). Alterations in Ca^2+^ homoeostasis and signaling are often associated with various pathological conditions, including cancer. The ROS regulatory system is closely linked to the Ca^2+^ signaling system which operates by changes in intracellular Ca^2+^ concentrations. Dysfunction of Ca^2+^ channels in the plasma or the mitochondrial membrane will disrupt the signaling system and increase ROS levels in any cell, potentiating harmful effects including cytotoxicity and resulting in pathogenesis of various disorders (44, 244, 321, 322, 328, 330, 331). Inversely, ROS can significantly affect calcium concentration in the cell by modifying the function of Ca^2+^ channels (233).

Increased levels of intracellular Ca^2+^ in some cases are associated with increased apoptosis, probably due to activation of Ca^2+^ dependent DNase I (332). This may be an alternative pathway for DNA damage and related pathologies. Changes in normal Ca^2+^ levels in the mitochondria can induce release of cytochrome C, a mitochondrial protein which is a signaling molecule for apoptosis in the cytoplasm, which then goes on to initiate apoptosis in the cell, and activation of nucleases which will cause DNA damage (330).

The effect of man-made EMFs, especially ELF, or RF pulsed or amplitude-modulated by ELF signals, on calcium concentrations in exposed cells and the unique role of the calcium VGICs or VGCCs in EMF-induced bioeffects have been well-documented for long time (8–10, 18, 22, 44, 238, 298, 314–316, 333–341), and explained by the IFO-VGIC mechanism (2, 5, 297, 305, 306). Dysfunction of VGCCs will cause alterations in the intracellular calcium concentrations, impairment of the Ca^2+^ signaling system and consequent ROS overproduction.

Walleczek (44) reviewed many studies showing effects of ELF EMFs on cells of the immune system revealing the critical role of intracellular calcium. But until that time, the site of interaction of EMFs with cells was unknown, even though the facts were pointing toward the calcium ion channels in the cell membranes as a most reasonable explanation. At the same time, Liburdy (315) in a series of pioneering experiments showed that calcium influx in lymphocytes which occurred within minutes after the onset of ELF EMF exposures, was due to an effect on the calcium channels in the cell plasma membranes (most of which are voltage-gated), and not due to release from intracellular stores.

Apart from the effect of EMFs on Ca^2+^, Na^+^, K^+^, etc. VGICs, proton (H^+^) VGICs will be similarly affected (342, 343). This in turn will disturb the function of NOXs triggering ROS generation (section 3.1.2). Thus, not only VGCCs, but all VGICs are the sites where the effects of man-made EMFs on cells take place (2, 210).

Besides many other adverse effects, ROS can also affect ion channels themselves. For example, many ion channels contain cysteine residues with highly reactive thiol (SH) groups. These are particularly susceptible to oxidation by ROS. Oxidation of cysteine residues can lead to formation of disulfide bonds. This can alter the channel conformation and affect channel gating. Another effect of ROS on VGICs can be the formation of sulfenic, sulfinic, or sulfonic acids: These modifications can change the channel’s structure and function, potentially leading to channel inactivation or altered ion permeability (318). Oxidation of K^+^ channels by ROS is a common event in the aging brain (344). Therefore, ion channel dysfunction leads to ROS overproduction, and ROS further amplify ion channel dysfunction. Obviously, we have a vicious circle here, where VGIC dysfunction leads to OS in cells, and this in turn disrupts the ion channels even more, leading to even more pronounced OS.

**Figure 5:**
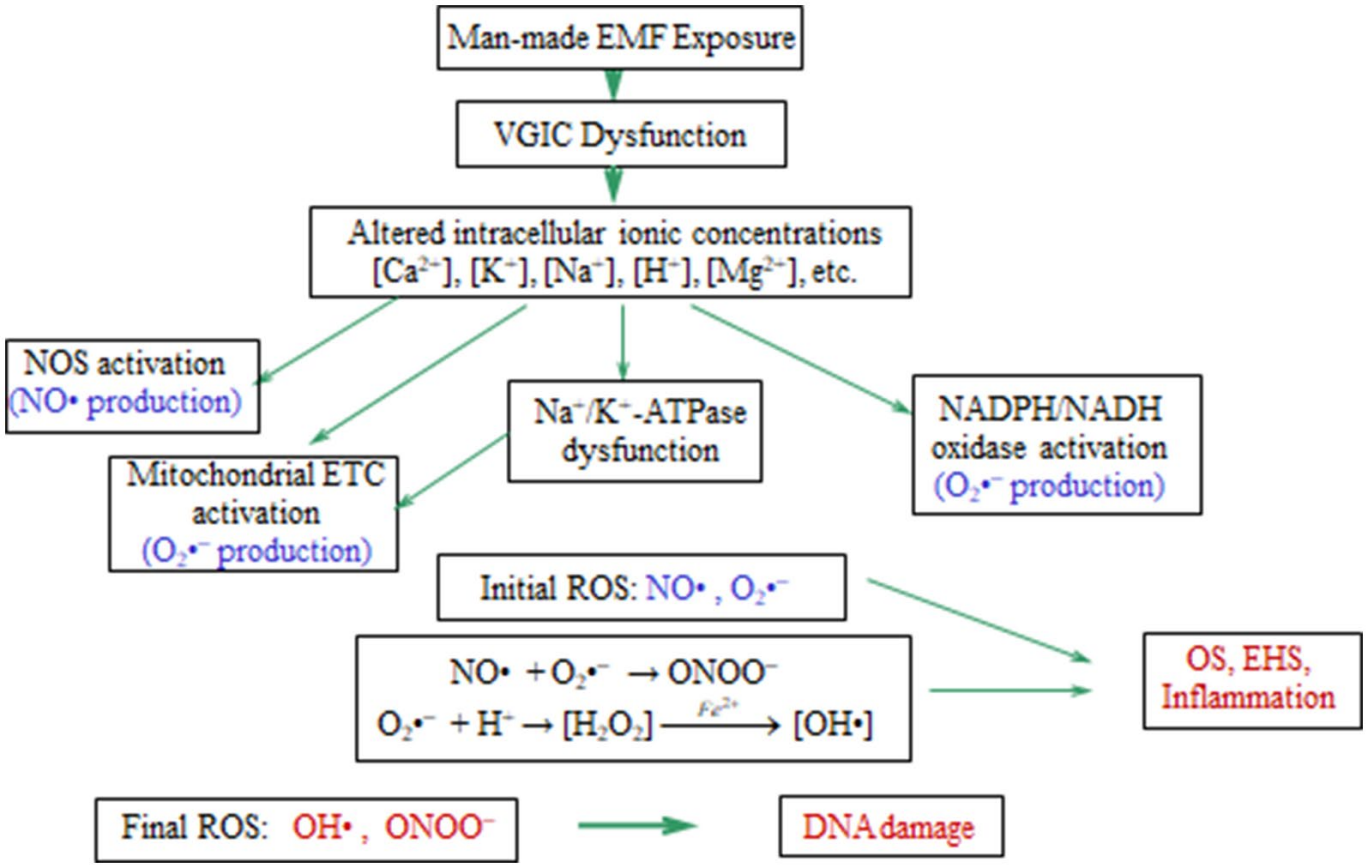
Schematic diagram of the OS processes initiated in living cells after anthropogenic EMF exposure and dysfunction of VGICs. Genetic/cell damage is executed by the generated “final” ROS [adapted from Panagopoulos et al. (210)].

The balance of the various mobile ions in a cell is closely linked to and, in fact, determines the cell’s homeostasis. ROS production in all cells initiates after imbalance of ion concentrations. Dysfunction of ion channels or pumps due to any reason, including EMF-exposure, can readily cause ionic imbalance, ROS overproduction and OS. Figure 5 shows the biochemical processes related with OS, and initiated after EMF-induced dysfunction of VGICs and imbalance of ion concentrations.

**Figure 6:**
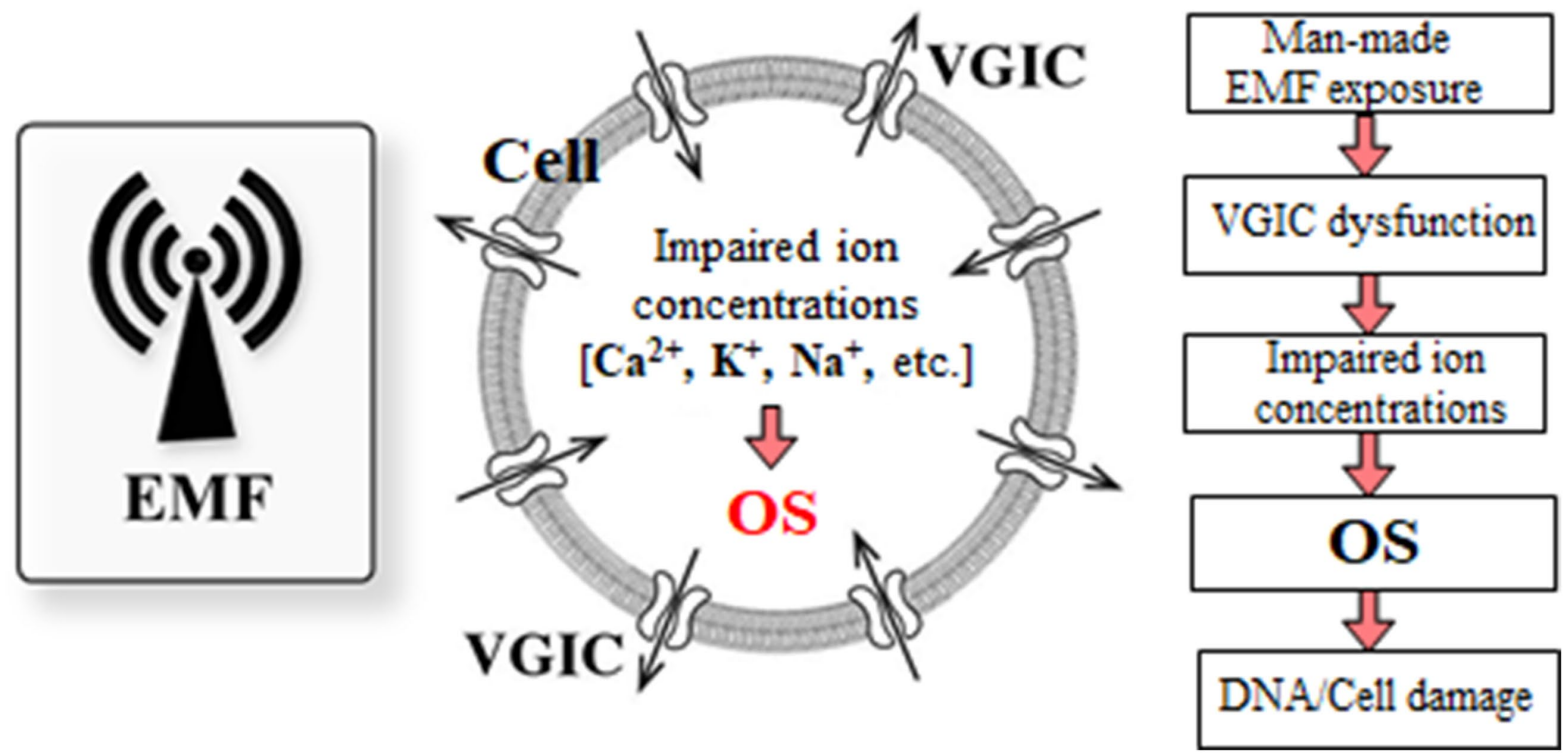
Comprehensive mechanism of anthropogenic EMF-induced bioeffects.

It is thus well documented that ion channel dysfunction causes OS, and here we make the case that the OS found after anthropogenic and especially WC EMF exposure is induced after VGIC dysfunction. We have a clear sequence of events starting from irregular gating of VGICs by man-made EMFs up to OS, cell/DNA damage and related pathologies including infertility and carcinogenesis. Therefore, a comprehensive mechanism of EMF-induced bioeffects can be clearly delineated, with a biophysical stage causing VGIC dysfunction and ionic imbalance, and a subsequent biochemical stage resulting in OS-related pathogenesis. Figure 6 shows a schematic representation of the described comprehensive mechanism initiated by EMF-induced dysfunction of VGICs, and resulting in OS and cellular damage.

## 6 Discussion

We have reviewed experimental and epidemiological studies referring to the biological and health impacts of anthropogenic ELF and WC EMF exposures. We find once again that it is well documented that both purely ELF and WC/RF (containing ELF) man-made EMFs induce OS and genetic damage, which can lead to related pathologies, such as infertility and cancer in both humans and animals.

We have documented that all anthropogenic EMFs referred to as “RF,” especially WC EMFs, apart from their RF emissions (carrier waves), emit ELF/ULF/VLF EMFs in the form of modulation, pulsation, and variability, and thus, in fact, are a combination of RF and ELF/ULF/VLF EMFs.

Some authors confuse “pulsation” with exposure intermittence. Zahumenska et al. (373) applied an intermittent exposure (6 × 10 min) to a continuous-wave LF EMF (87–207 kHz), with 10-min pauses between exposure periods, and claimed they studied effects of pulsed EMF, finding no significant difference from the absence of effects with an uninterrupted exposure (1 × 60 min). This is not the case. In a “pulsing field” the on/off pulsations are inherent in the signal and occur at ELF/VLF rates usually of the order of hundreds/thousands pulsations per second, whereas in the “intermittent” exposure as in this case, the field/signal is interrupted externally by a timer, or even manually by use of a switch. While pulsed EMFs are, in almost all cases, found to produce significantly greater effects than continuous-wave (non-pulsed) EMFs, an intermittent exposure to any EMF may produce smaller effects than an uninterrupted exposure to the same EMF when the intermittence interval is long enough (e.g., ≥10 min) to allow the exposed organism repair damages and/or adapt to the stressor (72). By confusing pulsation with intermittence one may draw completely misleading conclusions. Zahumenska et al. (373) actually found no effect by use of a continuous-wave LF EMF, which was expected, but they claimed the found no effect with a “pulsed” EMF. A definition of the various physical parameters of EMFs can be found in Panagopoulos and Margaritis (345) and Panagopoulos et al. (3).

We have explained that all man-made EMFs are fully polarized and coherent, with low-frequency (ELF/ULF/VLF) intensity variations in the vast majority of cases, meaning they possess net electric and magnetic fields oscillating (at ELF/ULF/VLF rates) in single directions and in phase. This condition induces parallel and coherent low-frequency forced oscillations of mobile ions and other charged/polar molecules in living tissues. The IFO-VGIC mechanism has described how such oscillations induce dysfunction of VGICs in the membranes of all cells resulting in altered intracellular ionic concentrations (2, 5, 297, 305, 306).

According to the IFO-VGIC mechanism, the non-thermal biological/health effects reported in the literature, are induced specifically by the low-frequency (ULF/ELF/VLF), and not the high-frequency (purely RF/MW), EMFs. This explains why, in the absence of low-frequency modulation/pulsation/variability, the non-thermal effects, attributed before to “RF” EMFs, disappear. It follows that purely RF/MW EMFs can only induce heating of biological tissues at adequately high intensities and frequencies approaching infrared (2, 3). An overview of VGIC structure and function, and the IFO-VGIC mechanism has been provided in section 4.

It is important to note that VGICs are not gated by direct forces on their S4 sensors by externally applied EMFs. That would require applied fields of the order of the transmembrane fields (~10^7^ V/m) (56). The reason why even very weak (down to 10^−5^–10^−4^ V/m) ULF/ELF anthropogenic fields can gate VGICs is that due to their polarized and coherent character combined with low frequency variability, they can induce parallel and coordinated low frequency forced oscillations of mobile ions within the channels. And the forces exerted on the S4 sensors by several oscillating ions in close proximity (≤1 nm), depending upon the inverse third power of the distance (Equation 12), are much greater than direct forces from externally applied EMFs. In other words, due to the IFO phenomenon in close proximity to the VGIC-sensors, the forces are enormously amplified. This is a key point in understanding the IFO-VGIC mechanism.

It is thus, polarization and coherence combined with low frequency variability that make anthropogenic EMFs able to irregularly gate (open or close) VGICs, the most sensitive EMF sensors and the most abundant class of ion channels in all cell membranes of all living organisms. This causes perturbation of ionic concentrations in the cells which in turn triggers (over)production of ROS. ROS can readily cause ionization/chemical alterations in living tissue, i.e., breakage of chemical bonds, and DNA damage.

We described biochemical processes initiated in living cells by dysfunction of VGICs due to man-made EMF-exposure, leading to altered intracellular concentrations of critical ions such as Ca^2+^, Na^+^, K^+^, H^+^, etc., and disruption of the cell’s electrochemical balance, redox state, and homeostasis. This leads to immediate production of the two initial ROS, superoxide anion (O_2_•^−^) and nitric oxide (NO•), which can then be easily converted to the powerful “final” ROS peroxynitrite (ONOO^−^) and/or hydroxyl radical (OH•), which can damage DNA or any other biological molecule.

It is remarkable that the same “final” ROS that ultimately cause biological damage in the case of EMFs (“non-ionizing” radiations), hydroxyl radical and peroxynitrite, are also found in the case of exposure to ionizing radiations. It is estimated that about 2/3 of the DNA damages caused by ionizing radiation are due to OH• (276, 294, 295, 346–348). This provides an answer to claims that “non-ionizing” anthropogenic EMFs cannot possibly cause biological damage. It comes that the same ROS that actually execute the biological damage in most cases, are produced by either ionizing radiation or “non-ionizing” EMFs/EMR. This is related with the fact that in most cases the action of radiation in biological tissue is indirect. The external agent causes impairment of cell homeostasis and in response, the cell generates ROS which execute the damage.

ROS sources in cells are the ETC in the mitochondria, the ETC in the NOXs in the plasma membrane, the NOS enzymes at various locations in the cell, and various other secondary sources (described in section 3.1). All ROS sources/promoters are affected by the intracellular concentrations of cations like Ca^2+^, K^+^, Na^+^, H^+^, with most cation channels being voltage-gated (VGICs) (231, 234, 271). Therefore, all ROS sources in cells can be affected by man-made EMFs. It is remarkable that in all cases reported so far in the literature, anthropogenic EMF exposures increase and not decrease ROS/OS in cells. This is an additional indication that the cells perceive anthropogenic EMFs as a disturbance.

Even though many of the details of the ion signaling that triggers ROS generation by the above sources are still unexplored, we do know that the triggering involves changes in the intracellular ionic concentrations. Since man-made EMFs have the ability to cause dysfunction of VGICs, the basic parts of the entire process leading to DNA damage and related pathologies are already identified, and the dots are already connected revealing the complete EMF-induced bioeffects mechanism.

Several questions still need to be addressed. For example, we did not discuss the state of the antioxidant system (AOS) under the condition of chronic OS due to long-term EMF exposure. As the production of ROS at physiological levels is an essential part in any cell’s life, the role of the AOS is to limit the level of ROS under the OS threshold where damage would ensue. Moreover, the AOS controls the activity of repair enzymes. Cells/organisms with compromised antioxidant capacity or high energy demands are particularly vulnerable to OS, and, subsequently, to man-made EMFs. Many studies have revealed significant changes in activity of key antioxidant enzymes under modulated and/or pulsed RF/WC EMF exposure [see reviews in (62–64)]. And while in many cases the changes in activities of antioxidant enzymes may be induced by ROS overproduction in the exposed cells, they may also be affected by ionic imbalances related to VGIC dysfunction. For example, Ca^2+^ can influence the activity of transcription factors like NF-κB and Nrf2, which regulate the expression of antioxidant genes (349). Further, disruption of Na^+^/K^+^ gradients can indirectly affect Ca^2+^ homeostasis through the Na^+^/ Ca^2+^ exchanger, which can operate in both directions depending on the ion gradients (350).

When overproduction of ROS in a cell exceeds the capacity of its AOS, the cell/organism is under OS. A sustained or repeated such condition leads to DNA/cellular damage. Intracellular ions, particularly Ca^2+^, affect the activity of AOS and DNA repair enzymes. For example, some DNA repair pathways are Ca^2+^-dependent. Disruption of Ca^2+^ homeostasis can therefore impair DNA repair capacity, making cells more susceptible to DNA damage (351). Unrepaired/misrepaired DNA lesions such as strand breaks, covalent bond breakage, or nucleotide base and sugar damages, can lead to cell senescence, cell death, or mutations, and related pathologies such as aging, infertility, neurodegenerative diseases, and cancer (2, 61, 210, 233, 280, 290).

The processes initiated in living cells due to VGIC dysfunction in their cell membranes resulting in OS, genetic damage, and related pathologies provide an explanation for the plethora of biological and health effects reviewed in the Introduction (section 1.2). Moreover, the dysfunction of VGICs caused by man-made EMF exposure and leading to OS can also explain EHS, as EHS is accompanied by OS (197, 352), and in fact it is likely due to chronic OS. The pathophysiological changes in the CNS observed to accompany EHS [see (190)] can be explained by the fact that neurons have higher percentages of VGICs, as VGICs (specifically Na^+^ and K^+^ VGICs) are the mediators for the transmission of the nerve impulses (231, 271).

Several studies have found that ELF EMFs induce epigenetic changes in cells, commonly resulting in altered gene expression. Such changes include methylation/de-methylation of genes via activation/deactivation of methyltransferase enzymes, post-translational modification of histone proteins, and alteration of microRNA expression (353–355). Epigenetic changes can induce significant alterations in cell function and consequently the health of an organism. Since ROS affect cell signaling (232) related also with epigenetic changes (320, 354), the reported epigenetic effects induced by anthropogenic ELF EMFs can be due to ROS signaling, and the presented mechanism of EMF-induced ROS (over)production provides an explanation for this. For example, EMF-induced ROS may interfere with DNA or histone methyltransferases and histone deacetylases, resulting in modifications of the epigenome at various regions, including the promoter regions of tumor suppressor genes, resulting in their silencing/inactivation, and leading to cancer promotion (356, 357).

Like us, Blank and Goodman (57, 58) also noted that both ELF and “RF” (actually WC) EMFs produce similar effects, especially in inducing synthesis of stress proteins in cells very rapidly (within a few min). For us, an apparent explanation of the common ELF and RF/WC EMF effects that escaped attention, is that “RF” EMFs affect cells not by their carrier (RF) components, but by their ELF components of pulsing and modulation. As this study shows, actually only the ELF EMFs are those that induce the non-thermal biological effects, and they do not act directly on DNA, but indirectly through VGIC dysfunction and consequent induction of OS (2, 210). Further, it follows that purely RF EMFs can only induce heating at adequately high intensities and frequencies (3).

As documented here, anthropogenic EMFs at environmentally existing levels can ionize living tissue through the action of the generated ROS/OS. It is through the action of ROS the damage found in the DNA after anthropogenic and especially WC EMF exposures. There is a tight link between anthropogenic EMF-exposures, VGIC dysfunction, OS, and DNA/cellular damage.

**Figure 7:**
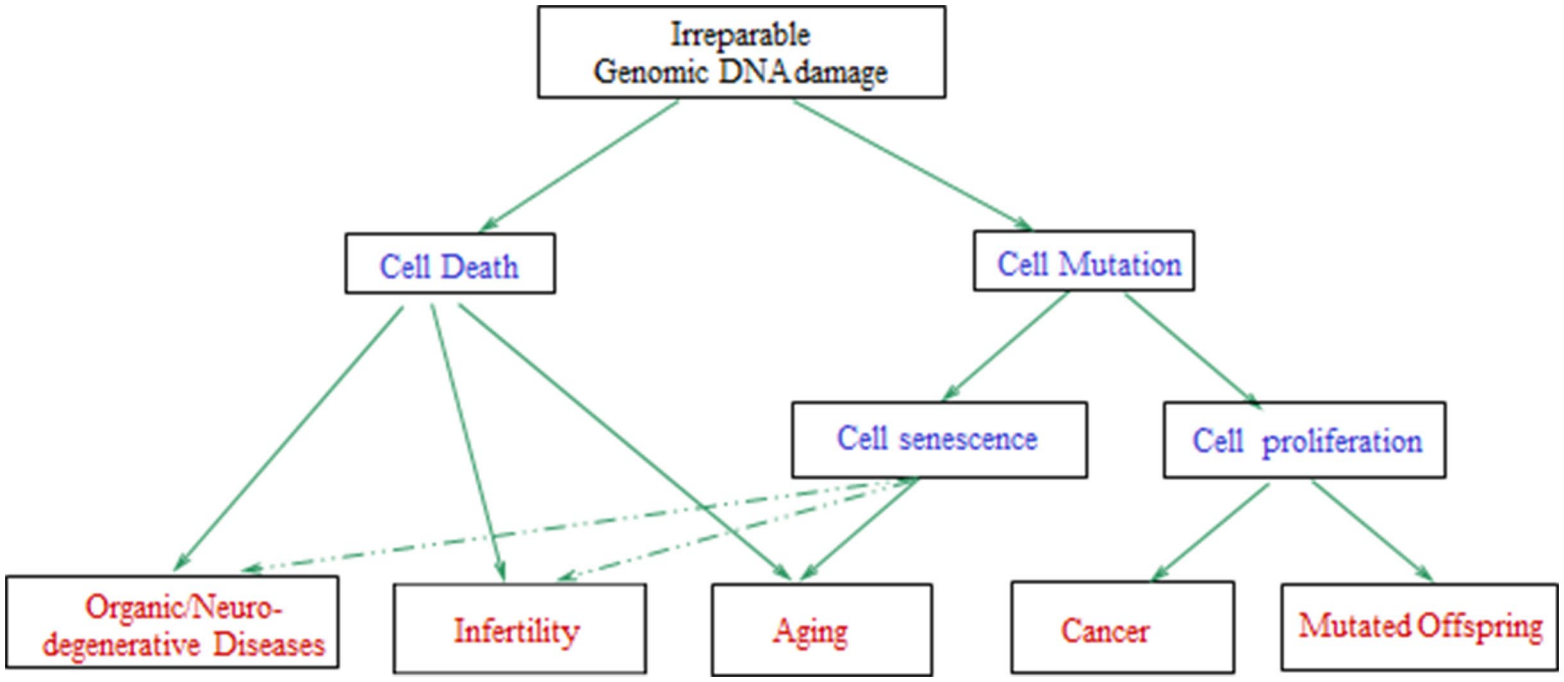
Schematic diagram with the possible consequences of irreparable genomic DNA damage in the cells of a living organism. The arrows with dashed lines mean that the effects can be induced in a lesser degree [adapted from Panagopoulos et al. (210)].

For cells with irreparably damaged genomic DNA, possible outcomes are, cell senescence or cell death (which may result in aging, organic/neurodegenerative diseases, and/or reproductive difficulties), cancer, or mutated offspring (Figure 7), depending on cell type, the specific biological/environmental conditions, and the state of the organism (2, 210). Thus, DNA damage induced by OS explains the pathologies linked to chronic exposure to anthropogenic EMFs, such as infertility and cancer.

Man-made EMFs, and especially the most detrimental ones from WC antennas/devices and high-voltage electric power lines, have become a new reality in modern life exposing billions of people on a daily basis (7, 50). Even though they are significantly less cytotoxic than radioactivity or certain toxic chemicals, they represent an evolutionary novel and most persistent daily cytotoxic agent, against which, existing repair mechanisms may not be efficient enough. Especially in individuals who are already genetically or epigenetically compromised.

Therapeutic effects of man-made EMFs have also been reported in the literature, especially of pulsing ELF EMFs and specifically in bone fracture healing (238, 358–363). Altered intracellular calcium levels have also been reported to accompany such effects, and the same biophysical mechanism of induced VGIC gating seems to be involved in both the detrimental and therapeutic effects of man-made EMFs (238, 359, 363). Several authors speak of therapeutic effects of pulsing ELF EMFs without specifying or discussing which parameter of the EMF exposure might be the therapeutic one (364–366). This might lead to the false impression that any EMF with ELF/ULF pulsations may be therapeutic, which, of course, is not the case, as, e.g., all WC EMFs consist of such pulsations, and yet are the most detrimental. Some other authors suggest that there are specific “beneficial” or “detrimental” frequencies in the ELF range (367) without considering the IFO-VGIC mechanism published for almost 25 years (and already referenced by more than a thousand other publications), which clearly shows according to generally accepted mathematics, physics, and biology, that the bioactivity of polarized, coherent, and oscillating EMFs, is proportional to field intensity and inversely proportional to field frequency, which makes all ULF/ELF frequencies very bioactive rather than only some specific ones (2, 5, 297, 305, 306). Thus, the basis of EMF bioactivity is not some specific frequencies, but polarization and coherence combined with low frequency variability (at any ULF/ELF frequency), with the lower the frequency the more bioactive the field (Equation 16). Once an EMF is polarized, coherent, and slow varying, we cannot exclude the possibility of resonance phenomena taking place at specific physiological ULF/ELF frequencies. However, we would not expect such phenomena, if they occur, to be particularly intense, especially under actual damping conditions within cells and ion channels (368).

In our opinion, a condition for an applied EMF in order to have a therapeutic action is, to simulate natural EMFs or physiological endogenous cellular signals. Once we know that the most bioactive polarized and coherent EMFs are the ULF/ELF ones, the critical issue for an applied ULF/ELF EMF is whether its included frequencies (and other parameters such as waveform, polarity, etc.) reinforce or cancel the endogenous physiological electrical activity of the cells which is responsible for the specific therapeutic action (49, 210, 369). The basic frequency of the natural atmospheric “Schumann” electromagnetic resonances (7.83 Hz) and its harmonics are detected in the human/animal brain activity, and the physical parameters of electromagnetic brain activity and atmospheric lightning display remarkable similarities (369–371). Thus, we have suggested (210) that the therapeutic effects of pulsed EMFs are expected to be optimal at pulsing frequencies coinciding with the Schumann frequencies, or the endogenous ionic oscillations in cells (49). Indeed, Yan et al. (372) found that pulses at an ELF repetition rate coinciding with the basic Schumann frequency 7.83 Hz inhibit proliferation and induce apoptosis of cancer cells while this does not occur with normal cells. This needs to be further verified and certainly, there are important limitations: All anthropogenic EMFs are fully polarized and coherent something that does not occur with the natural EMFs which are only partially polarized on certain occasions (5). This seems to be the reason why the vast majority of effects of anthropogenic EMFs are detrimental, whereas the vast majority of natural EMFs can be beneficial.

In conclusion, the IFO-VGIC mechanism that explains VGIC dysfunction, and the subsequent OS, provide a comprehensive biophysical/biochemical mechanism explaining the plethora of experimental and epidemiological findings connecting anthropogenic EMF exposures with OS, DNA/cellular damage and related pathologies such as poor health, EHS, infertility, organic/neurodegenerative diseases, cancer, etc. Even though the mechanistic details of how exactly the ionic perturbations stimulate ROS production by their sources need to be further explored, the basic scheme of the complete EMF-bioeffects mechanism is revealed already. The long existing experimental and epidemiological findings connecting exposure to man-made EMFs and DNA damage, infertility, and cancer, are now explained by the presented comprehensive mechanism. We hope this provides a better understanding of the involved science, a basis for future research, and the establishment of biologically relevant EMF exposure guidelines for effective protection of public health and the environment.

## Glossary

AOS: Antioxidant System
ATP: Adenosine Triphosphate
CNS: Central Nervous System
CYP: Cytochrome P450
DECT: Digitally Enhanced Cordless Telecommunications
DOX: Deoxyribose
EHS: Electro-hypersensitivity
ELF: Extremely Low Frequency
EMF: Electromagnetic Field
EMR: Electromagnetic Radiation
ETC: Electron Transport Chain
GEF: geoelectric field
GMF: geomagnetic field
GSM: Global System for Mobile telecommunications
IFO: Ion Forced Oscillation
LF: Low Frequency
MT: Mobile Telephony
MW: Microwave
NADH: Nicotinamide Adenine Dinucleotide
NADPH: Nicotinamide Adenine Dinucleotide Phosphate
NOS: Nitric Oxide Synthase
NOX: NADH/NADPH Oxidase
OS: Oxidative Stress
RF: Radio Frequency
ROS: Reactive Oxygen Species
SOD: Superoxide Dismutase
VGCC: Voltage-Gated Calcium Channel
VGIC: Voltage-Gated Ion Channel
VLF: Very Low Frequency
WC: Wireless Communication
Wi-Fi: Wireless Fidelity
ULF: Ultra Low Frequency
XO: Xanthine Oxidase
2G, 3G, 4G, 5G: Second, Third, Fourth, Fifth Generation of MT/WC
